# B Cells or T Cells in the Development and Sustenance of Rheumatoid Arthritis: Who is the Potential Contributor?

**DOI:** 10.31138/mjr.101025.rrw

**Published:** 2026-06-01

**Authors:** S Chandrashekara, Panchagnula Renuka, Pardhe Deepika

**Affiliations:** 1 ChanRe Rheumatology & Immunology Center & Research, Rajaji Nagar, Bengaluru, Karnataka, India;; 2 ChanRe Diagnostic Laboratory, Margosa Road, Malleshwaram, Bengaluru, Karnataka, India

**Keywords:** autoantibodies, T cells, B cells, synovium, germinal centres, inflammation

## Abstract

Rheumatoid arthritis (RA) is a systemic autoimmune disorder characterised by chronic joint inflammation driven by both cellular and humoral immune mechanisms. Autoantibodies and autoreactive lymphocytes play central roles in disease onset and progression. Although therapeutic strategies targeting T lymphocytes (T cells) and B lymphocytes (B cells) have benefited many patients, the precise cell type driving RA pathogenesis remains debated. In genetically predisposed individuals, immune tolerance is disrupted by environmental triggers such as smoking, infections, and stress. Antigen-driven adaptive immune responses dominate RA, with B cells undergoing clonal expansion and somatic hypermutation to produce high-affinity autoantibodies, notably anti-citrullinated protein antibodies (ACPA) and rheumatoid factor (RF). Synovial inflammation exhibits diverse lymphocyte infiltration patterns, including diffuse inflammation (~50%), T–B cell aggregates with germinal centres (GC)~24%, and aggregates without GC ~20%. T cells within synovial tissue often display clonal restriction, enriched in memory and effector subsets such as CD4^+^ Th17, CD8^+^ resident memory, and innate-like T cells (γδ, MAIT, and NK cells), all contributing to persistent synovitis. Dysregulated cytokine signalling, particularly through the JAK/STAT pathway, characterises active disease. In ACPA-positive RA, autoantibodies enhance inflammation via innate immune activation, while B cells also function as antigen-presenting cells (APCs) that sustain T cell activation. However, in some patients, synovial autoimmunity may occur independently of GC formation or prominent B cell involvement, underscoring the disease’s heterogeneity. This review highlights the evolving interplay between T and B cells from disease initiation to chronicity, which may help refine personalised immunotherapeutic approaches in RA.

## INTRODUCTION

While autoantibodies and autoreactive cells are implicated in rheumatoid arthritis (RA), their exact roles in sustaining inflammation remain unclear. Environmental triggers such as infections, smoking, pollution, and stress in genetically predisposed individuals are key factors that impair immune tolerance. Earlier studies have highlighted T lymphocytes (T cells) as central to RA pathogenesis.^1^ Following the success of anti-B cell therapy, there is significant interest in understanding B lymphocytes (B cells) role in autoimmune diseases. This review explores how both T and B cells contribute to disease development and heterogeneity.^2^ The initial trigger of autoimmunity in RA, whether mediated by T cells, B cells, or both, remains controversial. These cells differ in development, regulation, tolerance, and responses to autoantigens, and their involvement in RA initiation may influence clinical heterogeneity and varied therapeutic responses. As both cell types engage distinct mechanisms in immune defence, this review highlights their roles in clonal responses and regulatory pathways in RA pathogenesis. Cytokines and surface molecules further contribute to disease variability.^3^

## ANTIGEN, B CELL OR T CELL — THE CAUSE FOR PERSISTENCE OF INFLAMMATION?

Antigens drive adaptive immunity by shaping T- and B-cell responses. In RA, the persistence of chronic inflammation is attributed to a failure of tolerance to self-antigens, with ongoing diversification of B and T cell receptors (TCR). These processes differ between T and B cells, with B-cell development continuing throughout life.^4^ B cell maturation to memory or plasma cells is guided by B cell receptor (BCR) gene assembly, affinity, and antigen engagement in bone marrow and in its transitional stage via antigen-presenting cells (APCs).^5–7^ The majority of B cell maturation into plasma cells requires T cell assistance, although a small proportion of antigens can trigger B cell activation independently of T cells.^8^ In contrast, T cells develop in two phases in humans. Before the age of 20 to 25 years, they proliferate and enter circulation from the thymus. The thymic output declines after this age, and peripheral proliferation maintains the T cell pool.^9,10^ Upon infection or activation, T cells mature and evolve to a state of increased functional avidity, defined by their ability to respond to lower concentrations of peptide.^11–13^

Tolerance prevents harmful autoreactive T and B cells via central and peripheral checkpoints and autoimmunity develops when these mechanisms fail. Under normal circumstances, Antigen-driven clonal expansion later contracts, leaving memory cells for long-term immunity.^14^ The downgrading of immune response after antigen elimination differs between T and B cell populations. Memory B cells can persist in the absence of continuous T cell assistance for at least six weeks.^15^ In contrast, T cell responses are more dynamic; most effector T cells are eliminated after pathogen clearance, while a subset differentiates into long-lived memory cells.^16,17^ However, the majority of antigens targeted in RA persist, and antigenic stimulation continues to exist.

B cells play a multifaceted role in shaping T cell responses. They can activate experienced T cells while inducing tolerance in naïve T cells.^18^ Antigen presentation by B cells is essential for maintaining memory CD4^+^ T cells in a resting state, promoting their long-term survival.^19^ Various B cell subsets, including marginal zone and B1 B cells, contribute to early antibody responses and form part of the innate-like natural immune memory.^20^ In autoimmunity, long-lived plasma cells residing in niches such as the bone marrow sustain autoantibody production, thereby driving disease flares.^21^

A 25-year follow-up study by Amanna et al. demonstrated that memory B-cell counts do not correlate with antibody levels, indicating independent regulation. The type of antigen influences antibody longevity; for example, antibodies against Epstein-Barr virus (EBV) persist for life, whereas those against tetanus decline over the years.^22^ Experimental models using virus-like particles in mice showed that antibody levels are maintained according to plasma cell lifespan and antigen retention by follicular dendritic cells, rather than ongoing germinal centre (GC) activity. Cross-reactive or modified TLR signalling may also contribute to immune persistence.^23,24^ Understanding B- and T-cell development, interactions, and their roles in the regulation of autoimmunity is essential. Although T cell selection is better understood, it remains unclear whether B or T cells initiate autoimmunity, especially in diseases like RA.

## THE ROLE OF T CELLS

T cells are often considered the master regulators of the immune response.^25^ However, there are conflicting studies on the role of T cells in early stages of RA. Proposed hypotheses include dysregulated T-cell activation and differentiation as potential drivers of RA development.^26, 27^ Aslam et al. showed that IL-10 producing regulatory T cells (Tregs) can transition into IFN-γ producing cells, contributing to RA.^28^ Autoreactive T cells are present in both the synovium and peripheral blood of RA patients. While Tregs from healthy donors suppress these autoreactive cells, Tregs from RA patients exhibit impaired function.^29^ However, T cell profiles vary among individuals, leading to differing views on their clonality and role in RA pathogenesis.^30^

**Figure 1.**
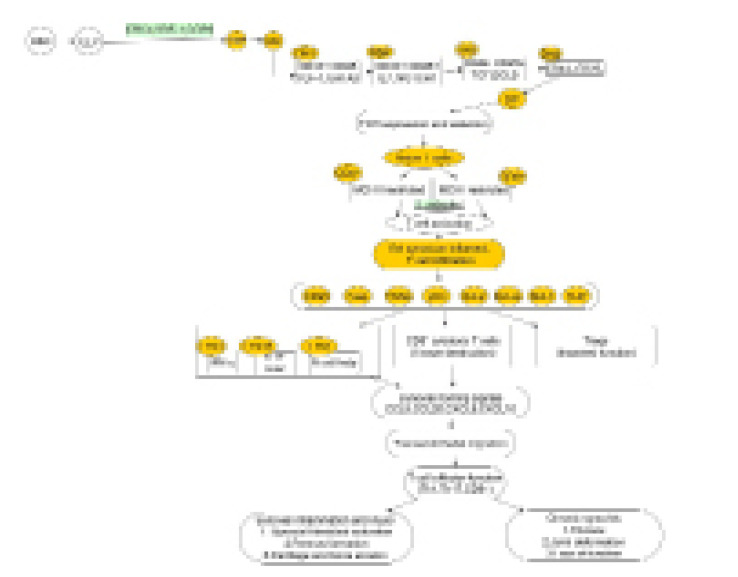
Illustration of T cell development and differentiation. HSC: Hematopoietic stem cell; CLP: Common lymphoid progenitor; TSP: T cell-specified progenitor; DN: Double negative (CD4^−^ CD8^−^) thymocytes; DN1: Double negative stage 1 (CD44^+^ CD25^−^); DN2:Double negative stage 2 (CD44^+^ CD25^+^); DN3: Double negative stage 3 (CD44^−^ CD25^+^); DN4: Double negative stage 4 (CD44^−^ CD25^−^); DP: Double positive (CD4^+^ CD8^+^) thymocytes; Th1: T helper 1 cells; Th17: T helper 17 cells; Thf: T follicular helper cells; Tregs: Regulatory T cells; CD4:Cluster of differentiation 4 (T helper cell marker); CD8:Cluster of differentiation 8 (cytotoxic T cell marker); CD25:Interleukin-2 receptor alpha chain; CD44:Homing/adhesion glycoprotein; CD95:Fas receptor (death receptor); TCR: T cell receptor; TCF-1: T-cell factor 1 (Wnt signalling); GATA3:GATA binding protein 3 (Th2 development); NOTCH1: Neurogenic locus notch homolog protein 1; TCF: T-cell factor; BCLB:B-cell lymphoma/leukaemia B; CRC4: C-C chemokine receptor 4; SDF-1: Stromal cell-derived factor 1 (CXCL12); CCR9: C-C chemokine receptor 9; CCL5:C-C motif chemokine ligand 5 (RANTES); CCL20:C-C motif chemokine ligand 20 (MIP-3α); CXCL9: C-X-C motif chemokine ligand 9 (MIG); CXCL10: C-X-C motif chemokine ligand 10 (IP-10); IL7:Interleukin-7 (T cell development); IFN-γ: Interferon-gamma (Th1 effector); IL17:Interleukin-17 (Th17 effector); IL22: Interleukin-22 (Th17 effector); FasL: Fas ligand (death ligand); TRAIL:TNF-related apoptosis-inducing ligand, p53:Tumor protein p53 (cell cycle checkpoint); Bcl-2: B-cell lymphoma 2 (anti-apoptotic); Bcl-xL: B-cell lymphoma-extra-large (anti-apoptotic); Mcl-1: Myeloid cell leukaemia 1 (anti-apoptotic); FLIP:FLICE-like inhibitory protein (anti-apoptotic); MHC I: Major histocompatibility complex class I (CD8^+^ T cell restriction); MHC II: Major histocompatibility complex class II (CD4^+^ T cell restriction); RA: Rheumatoid arthritis; L-selectin: Lymphocyte selectin (CD62L, lymph node homing).

Previous studies have reported a restricted T-cell repertoire and clonality in the synovial fluid and peripheral blood of RA patients, and similar patterns have been observed in monozygotic twins. The findings suggest that this may represent a primary, genetically influenced event rather than a secondary consequence of inflammation.^31–36^ Clonal alterations are differentially influenced by autoantibodies, with a strong association observed in the presence of ACPA, whereas this effect is not seen with rheumatoid factor (RF).^37^ Human study by Klarenbeek et al. and murine study by Khabouri et al. demonstrated increased clonality and reduced diversity in the early infiltrative stage of RA. As inflammation persists, an expansion in both clonality and diversity is observed.^38,39^ In murine remission models, Chang et al. noted that CD8^+^ TRM persist in previously inflamed joints and mediate site-specific flares. Similarly, human RA joints contain a comparable CD8^+^ TRM population, particularly in late-stage leukocyte-poor synovium.^40^ Brief dynamics of T cells development from thymus to synovium as discussed above is represented in **Figure 1**.

## FUNCTIONAL STATUS AND REGULATION OF T CELL DYNAMICS

T-cell infiltration of tissues during the early stages of inflammation is central to RA pathogenesis.^41,42^ While initial studies emphasised the role of CD4^+^ T cells, subsequent evidence has shown that CD8^+^ T cells and innate like T cells, including gamma delta (γδ) T cells, NK cells, and MAIT cells, also contribute. Vδ1 γδ T cells are enriched in RA synovium, similar to their distribution in the gut and skin.^43,44^ T cells promote synovitis by producing inflammatory cytokines, with RA previously viewed as a type IV Th1-mediated disease.^45^ Advances in T cell biology highlights their diverse regulatory and effector roles, influenced by genetics, autoantibodies, and disease stage. This explains the clinical variability, inconsistent findings, and evolving understanding of T cells in RA. Increased numbers of CD4^+^, CD8^+^, and NK cells have been observed both in the synovium of RA and osteoarthritis (OA). Cell distributions vary between patients, over the disease course, and with drug exposure. However, data on these dynamics remain limited.^46^ The distribution of phenotypes of CD4^+^ and CD8^+^ cells in the RA synovium is shown in **Tables 1** and **2**.^47,48^ In early RA, there is an expansion of C-X-C chemokine receptor type 5 (*PD-1^+^CXCR5^−^*) peripheral helper T (Tph) cells in circulation, which subsequently infiltrate the synovium.^49^ These cells, found near B cells, promote the accumulation of CXCR5^+^ T follicular helper cells (Tfh) cells. Brown et al. showed significantly higher programmed cell death protein 1 (PD-1^)hiCXCR5^−^ Tph cells in early RA synovium compared to OA, while PD-1^hiCXCR5^+^ Tfh cells remain similar between RA and healthy controls. Both Tph and Tfh cells are found in proximity to B cells and GC B cells, supporting early and sustained T–B cell interactions in RA.^50^ The relative ratio and interplay between Tph and Tfh cells appear to regulate B-cell maturation, follicle formation, and subsequent disease progression.^51^

**Table 1.** CD4^+^ cells involved in synovial infiltration.

**Cluster name**	**Differentially expressed genes**	**Frequency in SF**
*CXCL13^*high Tph	*TNFRSF18, LAG3, CXCL13*	+++
Central memory CD4	*LTB, ZFP36L2, KLF2*	±
Effector CD4	*CXCR3, TGFB1, KLRB1*	++
Treg	*FOXP3, IL2RA, TIGIT*	++
Cytotoxic CD4	*NKG7, GNLY, GZMH*	+
SESN3 CD4	*TNFAIP3, SLC2A3, CDC14A*	+
*CXCL13^*low Tph	*PTPN13, PRDM1, NEAT1*	+
Proliferating CD4	*STMN1, MKI67, TUBA1B*	+
Activated CD4	*CST3, HLA-DRA, HLA-DPA1*	+

CXCL13^high^/low Tph: T peripheral helper cells with high or low CXCL13 expression; Treg: Regulatory T cells; SF: Synovial fluid; TNFRSF18: Tumour necrosis factor receptor superfamily member 18; LAG3: Lymphocyte-activation gene 3; CXCL13: C-X-C motif chemokine ligand 13; LTB: Lymphotoxin beta; ZFP36L2: Zinc finger protein 36 C3H type-like 2; KLF2: Kruppel-like factor 2; CXCR3: C-X-C chemokine receptor type 3; TGFB1: Transforming growth factor beta 1; KLRB1: Killer cell lectin-like receptor subfamily B member 1; FOXP3: Forkhead box P3; IL2RA: Interleukin-2 receptor alpha; TIGIT: T cell immunoreceptor with Ig and ITIM domains; NKG7: Natural killer cell granule protein 7; GNLY: Granulysin; GZMH: Granzyme H; TNFAIP3: Tumour necrosis factor alpha-induced protein 3; SLC2A3: Solute carrier family 2 member 3; CDC14A: Cell division cycle 14A; PTPN13: Protein tyrosine phosphatase non-receptor type 13; PRDM1: PR/SET domain 1; NEAT1: Nuclear enriched abundant transcript 1; STMN1: Stathmin 1; MKI67: Marker of proliferation Ki-67; TUBA1B: Tubulin alpha-1B chain; CST3: Cystatin C; HLA-DRA/DPA1: MHC class II molecules; +/±/++/+++: Relative abundance in synovial fluid (low to high).

**Table 2.** Proportion of CD8^+^ cells in peripheral circulation.

**scRNAseq clusters**	**CyTOF clusters**	**Differentially expressed genes**	**Abundance**
*GZMK* *^+^* *GZMB* *^−^*	PD-1^+^HLA-DR^+^/^−^	*GZMK, NKG7*	Joint = PB
*GZMK*^+^*GZMB* low	PD-1^−^HLA-DR^++^	*IFNG, HLA-DRB1*	Joint > PB
*GZMK^−^GZMB* high	PD-1^−^HLA-DR^+^	*PRF1, GNLY*	Joint < PB
*GZMK^−^GZMB^−^* *(naive)*	PD-1^−^HLA-DR^−^	*CCR7, IL7R*	Joint < PB

scRNA-seq: Single-cell RNA sequencing; CyTOF: Cytometry by time-of-flight; DEGs: Differentially expressed genes; PB: Peripheral blood; Joint: Synovial fluid or tissue; *GZMK*: Granzyme K; *GZMB*: Granzyme B; PD-1: Programmed cell death protein 1; *HLA-DR*: Human leukocyte antigen – DR isotype; IFN γ: Interferon gamma; *HLA-DRB1*: Gene encoding a subunit of *HLA-DR*; PRF1: Perforin 1; *GNLY*: Granulysin; *CCR7*: C-C chemokine receptor type 7; *IL7R*: Interleukin-7 receptor; ^+^/^−^, ^−^, ^++^, etc.: Indicates expression levels (positive, low, high, etc.).

Within the CD4^+^ T-cell compartment, Tph cells are more active than other subsets and are highly enriched in the synovium compared with peripheral blood.^52^ The presence of prominent CD8^+^ T-cell population expressing granzyme K in the synovium suggests cytotoxic potential.^48^ The peripheral T lymphocyte phenotype in very early RA is not similar to that observed in clinically confirmed RA.^54^ Pandya et al. reported that in patients with early RA, the balance of circulating Th cells is skewed toward Th2 and Th17 phenotypes and away from the Th1 phenotype.^55^ Niu et al. also observed an increase in CD4^+^ T cells in such patients. The imbalance between Th17 cells and forkhead box p3 (Foxp3^+^)CD4^+^CD25^+^ Treg cells is driven by an expansion of Th17 cells and a reduction in Foxp3^+^CD4^+^CD25^+^ Treg cells.^56^ Studies evaluating the composition of T cells in predicting the onset of arthritis, particularly in patients with anti-CCP–positive antibodies, indicate a clear reduction of naïve T cells and Tregs in early RA.^57–59^

The expression of the *p53* gene and the secretion of granulocyte-macrophage cology-stimulating factor (GM-CSF) from synoviocytes and macrophages influence immune regulation. Synovial fibroblasts (SFib) upregulate B cell lymphoma-extra large (*Bcl-xL),* one of the anti-apoptotic proteins of the Bcl-2 family*,* in T cells through integrin–ligand interactions, thereby promoting T-cell survival.^59–60^ Elevated levels of anti-apoptotic proteins and activation of phosphoinositide 3-kinase/protein kinase B pathway (PI3K/Akt) pathway also suppress apoptosis.^61^ Aberrant integrin activation induces p38 mitogen-activated protein kianse (MAPK) phosphorylation, inhibiting *Fas*-mediated apoptosis.^62^ Additionally, stromal cell-derived factor 1 (SDF-1-CXCR4) interactions inhibit T cell apoptosis and promote their accumulation in the synovium.^63^

Impaired Tregs function reduces apoptosis.^61^ Berg et al. reported that T cells from patients exhibited a reduced TCR-dependent proliferative response following stimulation with anti-CD3 antibodies, along with diminished induction of IL-2 and IL-4. However, inerferon gamma (IFN-γ) induction was not defective when cells were stimulated with PMA/ionomycin, in contrast to the diminished IFN-γ response observed after anti-CD3 stimulation.^64^ Al-Janadi et al. noted altered cytokine profiles in RA patients compared to healthy controls, including increased IL-6 production. IL6 induced the expansion of a CD4^+^CD29^+^ T cell subset expressing TCR γδ antigens at sites of inflammation, which is implicated in immunopathology and autoantibody production.^65^ The clinical response to IL-6–targeted therapies underscore the central role of IL-6 in RA pathogenesis. CD4^+^ T cells in patients with RA show an altered cytokine secretion profile, producing reduced levels of IL2 and IFN-γ, but increased levels of IL4 and IL10, which are characteristic of a Th2-type immunoregulatory response. These changes are not limited to cytokine production; secondary signalling pathways, such as the JAK/STAT pathway, are also dysregulated in active RA but tend to normalise following effective treatment. In a study employing a systems immunology approach to simultaneously quantify 42 signalling nodes across 21 immune cell subsets, multiple differences were observed between patients with RA and healthy controls. Higher RA disease activity, compared to lower disease activity, was associated with either diminished or exaggerated responses to external stimuli across several immune cell subsets. Significant shifts were noted from pre-treatment to post-treatment following methotrexate or anti–tumour necrosis factor (anti-TNF) therapy. These shifts were most pronounced in patients who responded to treatment, with their immune signalling profiles moving closer to those observed in health controls.^66^

## TREG CELLS AND INFLAMMATION BALANCE

The balance between pro-inflammatory and counter-regulatory mechanisms is crucial for controlling the inflammatory response and its sequelae, particularly when antigen elimination is not possible. Tregs have been considered to play a central regulatory role in the development of RA and other autoimmune diseases.^67^ But there are conflicting findings on the impaired function and number of Treg cells present in the synovium of RA patients. An increased number of Treg cells is effective in reducing the inflammatory activity of synovial tissue cell cultures *ex vivo,* while reduced FoxP3^+^ Tregs have been noted in the synovium of RA patients compared to the peripheral blood.^68^ RA Synovial fibroblasts (RASFib) exerts a dual action on the equilibrium between Treg and T effector cells through its constitutive IL-15 expression. This results in altering the balance toward a proinflammatory state without reducing the number of cells either in the periphery or in the synovial fluid.^69^

The reduction in Treg cell number and function is attributed to persistent inflammation or a possible intrinsic defect, leading to IL-10 dominance and a shift toward a Th2-biased response. Studies evaluating the role of Treg cells and IL-10 have reported contradictory effects, depending on autoantibody status. In the presence of autoantibodies, increased TH2 skewing has been noted, resulting in increased inflammation.^70^ Thus, the presence of autoantibodies can alter the disease process. Recently, Cui et al. reported that Tregs cells can shift toward a Th17 phenotype under inflammatory pressure, particularly in the presence of IL6. The Th17/Treg ratio is often considered a key factor influencing the intensity of the inflammatory response.^71^ Several dysregulations in T cell physiology lead to persistent inflammation in the synovium, and heterogeneity in T cell populations, behaviour, and composition may contribute to variations in both clinical presentation and therapeutic response.^72^ The mechanism by which initial autoreactive T cells migrate to or infiltrate the synovium remains uncertain. However, the observed limited clonality of synovial T cells suggests specific localisation, and whether this limited clonality is a cause or consequence of disease remains unresolved.

## B CELLS AND AUTOIMMUNITY

Until the success of the anti-B cell therapy rituximab, the role of B cells in RA was considered relatively minor, limited mainly to antibody production. However, advances in B cell biology have revealed that B cells also play critical roles through antigen presentation, cytokine production, and regulatory functions, all of which contribute to shaping the inflammatory milieu. These insights highlight that B cells have a major role not only in initiating and maintaining immune responses against pathogens but also in the pathogenesis of autoimmune diseases. B cells are produced continuously throughout life from the bone marrow. Unlike T cells, B cells exhibit greater clonal diversity and have the unique ability to recognise antigens directly, without the need for APCs or costimulatory molecules. Furthermore, somatic hypermutation, which occurs after the initial repertoire selection, further enhances the diversity and adaptability of the B cell population.^73–75^

Tsuiji and colleagues proposed the presence of three checkpoints in the tolerance pathway of B cells, which reduce the autoreactive antibody repertoire. The first checkpoint is in the bone marrow and the second and third in the periphery.^76^ Wardemann et al. demonstrated that approximately 20% of antibodies produced by mature human B cells show reactivity to human epithelial cell line (HEp-2) antigens, and about 4.3% are polyreactive, potentially contributing to natural antibody production. However, the persistence of autoantibody-producing B cells has been frequently observed in diseases associated with tissue destruction.^77^ Tsuiji et al. noted the elimination of B cells expressing self-reactive and broadly reactive repertoire against bacteria during the transition from naïve to IgM^+^ memory B cells.^76^ Lang et al. and Halverson et al. suggested that receptor editing and anergy, rather than deletion, account for much of B cell tolerance as a third checkpoint.^78,79^

During an immune response, B cells specific for inciting antigen are activated and differentiated into plasma blasts before releasing into the circulation. Tan et al. sequenced the plasmablast antibody repertoire to identify the targets of the active immune response in RA and found that autoantibodies against α-enolase, citrullinated fibrinogen, and citrullinated histone H2B were produced by ongoing B cell activation.^80^ Bellatin et al. further noted the production of ACPA by cultured B cells derived from RA patients, as determined by reactivity to cyclic citrullinated peptide (CCP). Around 22% of the healthy subjects also had B cells that could produce ACPA. Patients with HLA-DR alleles carrying the RA-associated shared epitope had more B cells with autoimmune potential for CCP than those without the HLA alleles (odds ratio 8.1, P = 0.001).^81^

**Figure 2.**
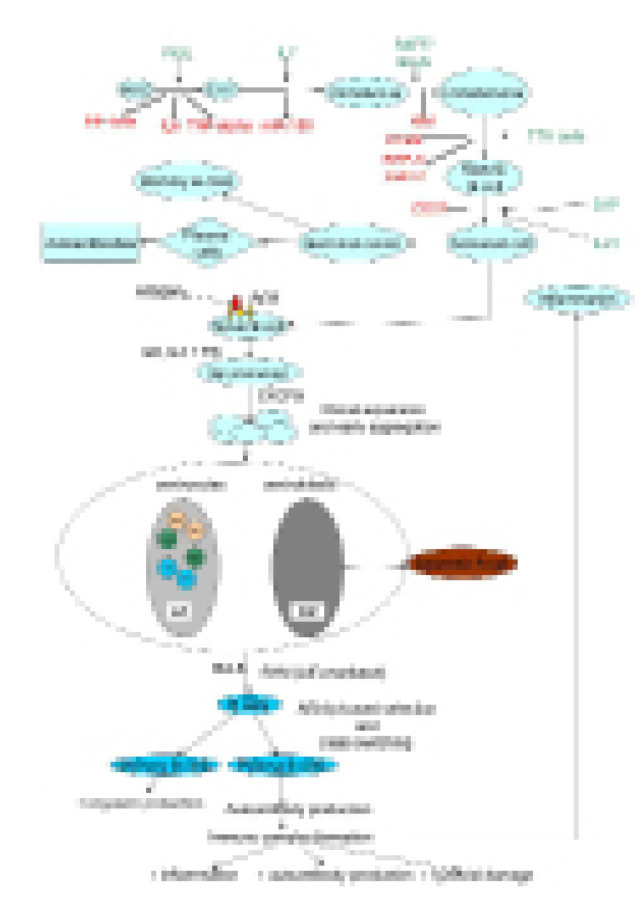
Illustration of B cell development and autoimmune-mediated synovial tissue damage. HSC: Hematopoietic stem cell; CLP: Common lymphoid progenitor; TFH:T follicular helper cells; Gc: Germinal centre; LZ: Light zone (of germinal centre); DZ :Dark zone (of germinal centre); Flt3L: Fms-like tyrosine kinase 3 ligand; BAFF: B-cell activating factor; BLyS: B-lymphocyte stimulator; PTEN: Phosphatase and tensin homolog; SHP-1: Src homology region 2 domain-containing phosphatase-1; SHP-2:Src homology region 2 domain-containing phosphatase-2; Bcl-6:B-cell lymphoma 6 protein; BCR: B cell receptor; IL7:Interleukin-7; IL6:Interleukin-6; IL4:Interleukin-4; IL21: Interleukin-21; TNF-alpha: Tumour necrosis factor-alpha; INF-beta: Interferon-beta; miR-150: Microrna-150; S1P: Sphingosine-1-phosphate; CD22: Cluster of differentiation 22; CXCR5: C-x-c motif chemokine receptor 5; BIM :Bcl-2-like protein 11 (pro-apoptotic protein); SHM: Somatic hypermutation; AID: Activation-induced cytidine deaminase; ↑:Increased/upregulated; ↓ : Decreased/downregulated.

Normal synovium contains very few B cells, but their numbers increase significantly with inflammation. Under Tfh cells and IL-6 influence, they form GCs, undergo maturation, and somatic hypermutation (Fig. 2). Higher B cell infiltration has been shown to correlate with serum anti-CCP and RF positivity.^82^ Synovial B cells also demonstrate substantial clonal expansion, with age-associated B cells (ABCs), memory B cells, and activated B cells clonally linked to synovial plasma cells. Among these, non-naïve subsets such as ABCs, *NR4A1*^+^ activated B cells, and plasma cells are enriched in the synovium and display higher rates of somatic hypermutation compared with B cells in peripheral blood.^51^

In addition to antibody production, B cells have several other key functions, including cytokine secretion, antigen presentation, and interactions with T cells and dendritic cells. B cells have been shown to produce cytokines such as IL-4, IL-6, IL-10, IFN-γ, transforming growth factor-β, and lymphotoxin-α ^83^ However, the production of several cytokines by B cells upon BCR engagement is suppressed. Additionally, RA B cells exhibit reduced expression of the pro-apoptotic factor TRAIL compared with healthy donor B cells. These defects are partially reversed following treatment with tocilizumab, indicating that abnormal IL-6 signalling contributes to them. The role of B cells as regulatory cells in autoimmunity has received significant attention in recent years.^84,85^

Though their role has not been clearly elucidated, studies suggest that eliminating B cells, which may often include regulatory B cells, may produce opposite effect.^86,87^ Regulatory B cell function has been reported to be impaired in RA.^88,89^ Some studies indicate a reduction in the number of these cells; however, their role in regulating disease progression and development requires further clarification. B cells can trigger auto-immunity through incomplete deletion of autoreactive cells, and somatic hypermutation (SHM) can subsequently contribute to the maturation and diversification of the immune response. These attributes suggest that B cells play a critical role in driving RA and contributing to its evolving heterogeneity (**Figure 2**). The aberrant B cell alterations observed in patients with RA^90^ is summarised in **Table 3**.

**Table 3.** Summary of circulating aberrant B cell subsets and their clinical relevance in autoimmune diseases.

**B subset**	**Stage**	**Extrinsic and/or intrinsic mechanism**	**Relevance to the diseases**
CD21^−^/low B cells ↑	Naïve and memory B cells	Increases B cell activation	Correlates with lymph proliferation
CD86^+^ B cells ↑	Activated B cells	Possible association with inducible T- cell costimulator (ICOS^+^) Tfh cells and serum IL-21	Elevated levels associated with disease severity
IgD^−^CD27^+^ memory B cells ↑	Memory B cells	------	Correlates with disease activity and the anticyclic citrullinated protein antibodies
IgD^+^CD27^+^ memory B cells ↓	Memory B cells	Impaired IgM-production capacity and altered BCR repertoire	-------

CD: Cluster of differentiation (surface markers used to identify cell types); CD21^−^/low B cells: B cells with low or no expression of CD21 (complement receptor 2); CD86^+^ B cells: B cells expressing CD86, a costimulatory molecule involved in T cell activation; ICOS^+^ Tfh cells: Inducible T-cell costimulator positive follicular helper T cells; IL-21: Interleukin-21, a cytokine produced mainly by Tfh cells that promotes B cell activation and differentiation; IgD^−^CD27^+^ memory B cells: Class-switched memory B cells lacking IgD but expressing CD27; IgD^+^CD27^+^ memory B cells: IgM^+^IgD^+^ non-switched memory B cells; BCR: B cell receptor; ↑: Increased or elevated levels; ↓: Decreased or reduced levels.

## AUTOANTIBODIES AND HETEROGENEITY

While ACPA and RF are key biomarkers in RA pathogenesis, their exact role in early disease development remains unclear. Autoantibodies often appear months to years before clinical onset, suggesting that B cells may contribute to disease initiation, though only in a subset of patients. Seropositive and seronegative RA differ in phenotype, immunopathology, and treatment response. Approximately 70% of patients with established RA have autoantibodies such as RF and ACPA, underscoring their relevance in disease progression and their potential as early diagnostic markers.^91,92^ Sokolove et al. showed that RA patients harbour autoantibodies targeting innate immune ligands (e.g., histones, fibrinogen, biglycan) along with elevated cytokines (TNF-α, IL-6, IL-12p70, IFN-γ), pointing to a pre-clinical phase characterised by diverse autoantibodies and early epitope spreading.^93^ As the disease evolves, most patients switch to IgG anti-CCP2 isotypes, reflecting dynamic immune maturation.^94^ These observations indicate a progressive isotype shift and highlight the heterogeneity of the pathogenic process. RA heterogeneity begins even in the preclinical stage, marked by epitope spreading and broad ACPA cross-reactivity, with >70% of ACPA reacting with filaggrin among multiple citrullinated peptides (PAD, vimentin, enolase, fibrin, collagen-II, filaggrin). Goules et al. further confirmed this heterogeneity as a clinical feature, highlighting varied autoimmune responses in RA.^95^ In an earlier study, the author established the role of ACPAs in the onset, progression, and clinical heterogeneity of RA.^96^ Autoantibody levels and patterns differ among RA patients, with the highest titres of anti-MCV, anti-CCP, and RF observed in those with severe extra-articular manifestations such as Felty’s syndrome. The isotype of autoantibodies, whether RF or ACPA, has distinct roles in immunopathogenesis. The IgM ACPA response displays a more restricted antigen recognition profile than IgG ACPA (OR = 0.26, P < 0.0001).^97^ IgM RF has been shown to be a stronger predictor of disease severity, with the highest sensitivity (75%) compared to anti-CCP antibodies (68%) and Anti-keratin antibodies [AKA (46%)]. IgM RF positivity correlates with both clinical manifestations and severity of erosions.^98^

Clinical and genetic data suggest that ACPA-positive and ACPA-negative RA represent distinct disease subsets.^99^ACPA specifically bind to peripheral blood mononuclear cells (PBMCs) from CCP-positive RA patients via their Fab region and significantly enhance the expression of pro-inflammatory cytokines, with IL-1β and IL-6 mRNA levels increasing 10- and 6-fold, respectively, compared to control IgG. This effect is inhibited by about 30% in the presence of multiepitope citrullinated peptide (Cit-ME), a natural ACPA ligand. These findings indicate that the isotype and antigen specificity of antibodies such as ACPA can sustain autoreactivity and contribute to the clinical and immunopathological heterogeneity observed in RA.^100^

Neutrophils and the neutrophil extracellular traps (NETs) they generate are an important source of citrullinated proteins, producing citrullinated peptides that serve as key targets for ACPA in RA. Khandpur et al. showed that NETosis is markedly increased in RA neutrophils, leading to the externalisation of citrullinated autoantigens such as histones and vimentin. Elevated NET formation correlates with ACPA positivity and systemic inflammation, indicating that RA neutrophils are primed to release PAD-modified citrullinated proteins that link innate immune activation to adaptive autoimmunity. NETs also amplify inflammation by stimulating synovial fibroblasts and promoting pathogenic processes within the joint.^101^ Wu et al. further demonstrated that ACPAs can themselves enhance NET formation, creating a self-perpetuating cycle in which NETs activate fibroblast-like synoviocytes and intensify synovial inflammation.^102^ Additionally, Ribon et al. reported that ACPAs preferentially recognise NET-derived antigens rather than those from resting neutrophils, supporting the role of excessive NETosis and NET activity in driving RA pathogenesis at multiple levels.^103^

GC-positive follicles in the synovium show B-cell proliferation and dendritic cell networks, supporting affinity-matured autoantibody production. These ectopic GCs facilitate B-cell activation, class switching, and interaction with T cells, thereby perpetuating chronic inflammation. In addition, B cells act as APCs that activate T cells. Studies have demonstrated an association between synovial GCs and the maturation of RF.^104,105^

However, other findings suggest that lymphoid neogenesis in RA is reversible and not always essential for sustaining local autoimmunity, reflecting heterogeneity in synovial pathology.^106^ The recent R4RA trial evaluated synovial infiltration patterns and reported a significant association between histopathological subtypes and therapeutic response, suggesting that B-cell biology contributes to heterogeneity in treatment outcomes.^107^ Both B cells and CD8 T cells are essential for the formation and maintenance of ectopic GCs in rheumatoid synovium. B cells are required for T cell activation, while CD8 T cells contribute critically to overall GC function.^108^ In depletion experiments, removal of synovial B cells abolished T-cell responses, and non-B-cell APCs were unable to sustain T-cell activation. Moreover, GC function in rheumatoid lesions was unexpectedly dependent on CD8 T cells, with TCR sequences from CD8 T cells shared across distinct GCs.^109^ Treatment with TNF-α inhibitors and rituximab has been shown to disrupt and dissolve ectopic GCs in RA synovium.

## T CELL OR B CELL?

B-cell subsets contribute to RA flares through distinct pathways. For example, short-lived plasma cells can arise in a T-independent manner, reside locally in inflamed tissue, and continue antibody secretion despite anti-proliferative therapy, in contrast to T-dependent memory B cells and long-lived plasma cells.^110^

Research has also highlighted key T cell features in RA, including skewed V gene usage and preferential clonal expansion in synovium compared with peripheral blood. However, similar TCR alterations are observed in other inflammatory joint diseases, indicating these changes are not RA specific. ACPA positive and ACPA negative RA differ at the molecular level, reflecting distinct T cell involvement. Smoking and high anti-CCP titres correlate with more aggressive disease, while clinical severity is further influenced by anti-CCP isotype and epitope specificity. Epitope spreading, associated with early antibody production and affinity maturation, often precedes clinical onset. B cells contribute to these processes by presenting antigens and producing cytokines that regulate and amplify immune responses.^111,112^

T cells alone cannot sustain antigen-triggered inflammation due to limited antigen access and reliance on major histocompatibility complex (MHC)-mediated presentation. B cells, through the BCR and fragment crystallisable gamma (Fcγ) receptors, efficiently capture, process, and present antigens, particularly low-affinity ones, thereby enhancing T cell responses. In the absence of B cells, T cell activation diminishes. While dendritic cells and other APCs contribute to antibody-dependent cytotoxicity and cellular immunity, B cells are critical for maintaining T cell responses. T cell regulation in RA is reflected in the altered Th1/Th17 ratio, which inversely correlates with disease activity. Both B and T cells influence RA pathogenesis differently across individuals, contributing to disease heterogeneity.

The cell type involved in the initiation of the autoimmune process is a matter of debate. Evidence supports roles for both B and T cells, and it is reasonable to assume that dysfunction in both cell types may be necessary for disease onset. Clinical observations further suggest that RA may comprise distinct subsets, with patients who have low autoantibody titres representing one entity and those with high titres often exhibiting more aggressive disease. This raises the possibility of T cell–dominant (Th1) RA and B cell–dominant (Th2) RA, with potential for dynamic interplay or switching between these phenotypes.

## EVIDENCE FROM T AND B CELL-BASED THERAPEUTIC INTERVENTIONS

Targeted immunotherapies have transformed the treatment of autoimmune diseases by modulating T and B cell pathways. However, there have been limited success stories with T cell–directed interventions. Alemtuzumab, an anti-CD52 antibody that depletes both T and B cells, has shown efficacy in multiple sclerosis but is limited by profound immunosuppression. In contrast, broad T cell–depleting strategies such as anti-CD4 therapies failed due to excessive immunosuppression and infection risk without clinical benefit. Long-term challenges, including T cell exhaustion and senescence, further limit durable responses.^113^ Abatacept (CTLA-4 Ig) blocks the CD28–CD80/86 co-stimulatory signal, suppressing autoreactive T cells and reducing inflammation in RA.^114^

In contrast, B cell–targeted strategies have been more successful. Rituximab (anti-CD20), which depletes B cells, improves outcomes in RA and systemic lupus erythematosus (SLE).^115^ Belimumab, an anti-BAFF antibody, reduces B cell survival and lowers autoantibody production and has been a success in SLE, though it failed to show significant improvement over conventional therapy in some studies.^116^ More recently, CD19-directed CAR T cells have shown promise in refractory autoimmune diseases such as lupus and systemic sclerosis, but their role in RA remains to be explored. Emerging modalities such as CAR T cells and bispecific antibodies offer opportunities for safer and more personalised treatment approaches.^117^

Anti-cytokine strategies have been highly successful in RA. Inhibitors targeting TNF and IL-6 have proven effective, whereas blockade of IL-17 and interferon pathways has shown limited benefit.^118^ Broad cytokine blockade through the JAK–STAT pathway has also been useful in RA but demonstrated limited efficacy in SLE.^119^ Targeted therapies for RA vary in effectiveness. TNF, IL-6, and JAK inhibitors are most effective, while many B-cell, T-cell, cytokine, and signaling-pathway agents have failed due to limited benefit, toxicity, or safety concerns (**Table 4**).^120–146^

**Table 4.** List of biologics and targeted therapies used in RA.

**Component category**	**Pathway**	**B cell impact**	**T cell impact**	**Overall efficacy/failure reason**
**B-cell depletion agents**				
Rituximab	B cell	B-cell depletion	Minimal	Rituximab effective (ACR50: 27–34%), others failed due to no added benefit or safety
Ocrelizumab				
Ofatumumab				
**B cell proliferation inhibitors**				
Fenebrutinib	BTK / BCR Signalling	B-cell modulation	Minimal	Fenebrutinib: Comparable to adalimumab (Ph II/III); Evobrutinib: Mild GI AEs (Ph II);
Evobrutinib				
**T-cell targeting agents**				
Anti-CD4	T cell	Minimal to depletion	Variable: Blockade or Depletion	failure; others failed due to Treg loss, toxicity
Anti-CD5				
Alemtuzumab				
Abatacept	T cell	Minimal to depletion	Variable: Blockade or Depletion	Abatacept effective in DMARD/TNFi failure
**Cytokines**				
Anakinra	IL-1	Minimal	Minimal	Moderate efficacy (ACR20: ~45%), less effective than TNF inhibitors
Secukinumab	IL-17/IL-17R	Minimal	Minimal	Modest efficacy (ACR20 ~35%), not superior to existing therapies
Ixekizumab				
Brodalumab				
Adalimumab	TNF-α	Minimal	Minimal	High efficacy (ACR20: 55–75%), radiographic protection, rapid onset
Etanercept				
Golimumab				
Sarilumab	IL-6	Minimal	Minimal	Effective as monotherapy, superior to adalimumab (ACR20: 56–74%)
Ustekinumab	IL-12/23	Minimal	Minimal	Failed due to low p40 expression or poor clinical response
Sifalimumab	IFN-α	Minimal	Minimal	Inconclusive results; development redirected to SLE
Anifrolumab				
Mavrilimumab	GM-CSF	Minimal	Minimal	Mavrilimumab showed efficacy but was discontinued; Namilumab failed to meet benchmarks
Namilumab				
**JAK inhibitors**				
Tofacitinib	JAK-STAT	Broad suppression	Yes	ACR20: 57–75%, oral administration, superior to adalimumab in some cases
Baricitinib				
Upadacitinib				
**TsDMARD (p38 MAPK inhibitor / Syk inhibitor / TYK2 inhibitor)**				
Losmapimod	MAPK cascade	Minimal	Minimal	Modest efficacy in phase II; development discontinued due to insufficient benefit
Fostamatinib	Syk / FcR / BCR	B-cell signalling	Minimal	Previously approved

ACR20: American College of Rheumatology 20% improvement criteria; ACR50: American College of Rheumatology 50% improvement criteria; Ph II : Phase II (clinical trial); Ph III - Phase III (clinical trial); BTK: Bruton's tyrosine kinase; BCR:B-cell receptor; GI: Gastrointestinal; AEs: Adverse events; DMARD: Disease-modifying antirheumatic drug; TNFi: Tumour necrosis factor inhibitor; Treg: Regulatory T cells; IL-1: Interleukin-1; IL-17: Interleukin-17; IL-17R: Interleukin-17 receptor; TNF-α: Tumour necrosis factor-alpha; IL-6: Interleukin-6; IL-15: Interleukin-15; IL-21: Interleukin-21; IL-12: Interleukin-12; IL-23: Interleukin-23; IFN-α: Interferon-alpha; SLE: Systemic lupus erythematosus; GM-CSF: Granulocyte-macrophage colony-stimulating factor; OSM: Oncostatin M; JAK-STAT: Janus kinase-signal transducer and activator of transcription; Ts DMARD: Targeted synthetic disease-modifying antirheumatic drug; p38 MAPK : p38 mitogen-activated protein kinase; Syk: Spleen tyrosine kinase; TYK2: Tyrosine kinase 2; FcR: Fc receptor.

## CONCLUSION

Current evidence supports roles for both B and T cells, although one may dominate without being exclusively responsible. Initial errors in B cell regulation may trigger autoimmunity in the majority of RA patients. In addition to B cells, concurrent T cell sensitisation and active participation are required. Further disease propagation may be driven by either B or T cells. Identifying the dominant cell type and the key steps that sustain disease activity under specific circumstances could help guide appropriate therapeutic interventions and personalisation of biologics or disease-modifying antirheumatic drug (DMARD) choice.
